# Risk assessment of thromboembolic events in hospitalized cancer patients

**DOI:** 10.1038/s41598-021-97659-9

**Published:** 2021-09-14

**Authors:** Federico Nichetti, Francesca Ligorio, Giulia Montelatici, Luca Porcu, Emma Zattarin, Leonardo Provenzano, Andrea Franza, Luca Lalli, Filippo de Braud, Marco Platania

**Affiliations:** 1 Medical Oncology Department, Fondazione IRCCS Istituto Nazionale dei Tumori, Via Venezian 1, 20133 Milan, Italy; 2 Computational Oncology, Molecular Diagnostics Program, National Center for Tumor Diseases (NCT) and German Cancer Research Center (DKFZ), Im Neuenheimer Feld 280, 69120 Heidelberg, Germany; 3 Laboratory of Methodology for Clinical Research, Oncology Department - Istituto di Ricerche Farmacologiche Mario Negri, IRCCS, Milan, Italy; 4 Unit of Immunotherapy of Human Tumors, Fondazione IRCCS Istituto Nazionale dei Tumori di Milano, Milan, Italy; 5 Department of Oncology and Hemato-Oncology, University of Milan, 20122 Milan, Italy

**Keywords:** Cancer, Physiology, Oncology, Risk factors

## Abstract

Hospitalized cancer patients are at increased risk for Thromboembolic Events (TEs). As untailored thromboprophylaxis is associated with hemorrhagic complications, the definition of a risk-assessment model (RAM) in this population is needed. INDICATE was a prospective observational study enrolling hospitalized cancer patients, with the primary objective of assessing the Negative Predictive Value (NPV) for TEs during hospitalization and within 45 days from discharge of low-grade Khorana Score (KS = 0). Secondary objectives were to assess KS Positive Predictive Value (PPV), the impact of TEs on survival and the development of a new RAM. Assuming 7% of TEs in KS = 0 patients as unsatisfactory percentage and 3% of as satisfactory, 149 patients were needed to detect the favorable NPV with one-sided α = 0.10 and power = 0.80. Stepwise logistic regression was adopted to identify variables included in a new RAM. Among 535 enrolled patients, 153 (28.6%) had a KS = 0. The primary study objective was met: 29 (5.4%) TEs were diagnosed, with 7 (4.6%) cases in the KS = 0 group (NPV = 95.4%, 95% CI 90.8–98.1%; one-sided p = 0.084). However, the PPV was low (5.7%, 95% CI 1.9–12.8%); a new RAM based on albumin (OR 0.34, p = 0.003), log(LDH) (OR 1.89, p = 0.023) and presence of vascular compression (OR 5.32, p < 0.001) was developed and internally validated. Also, TEs were associated with poorer OS (median, 5.7 vs 24.8 months, p < 0.001). INDICATE showed that the KS has a good NPV but poor PPV for TEs in hospitalized cancer patients. A new RAM was developed, and deserves further assessment in external cohorts.

## Introduction

Thromboembolic events (TEs) represent a major complication in cancer patients, resulting in worsened quality of life and poorer prognosis. Indeed, thromboembolism is the second leading cause of death among patients with cancer^1^. Thromboprophylaxis (TP) with low molecular weight heparins (LMWHs)^2^ and Direct Oral AntiCoagulants (DOACs) was demonstrated to approximately halve TEs risk in ambulatory cancer patients^3,4^; however, anticoagulation brings a significant risk of bleeding complications. Therefore, several TEs risk assessment models (RAMs) have been developed in order to stratify cancer patients and provide a tailored prophylaxis only to those who are at higher TEs risk, while sparing low risk patients from an unjustified risk of bleeding. The most widely used risk stratification tool is the Khorana Score (KS), which groups patients in risk categories based on tumor type, blood count and body mass index (BMI)^5,6^. Several other RAMs testing different variables were designed, with heterogeneous results and sometimes modest applicability in routine clinical practice^7^. However, all these RAMs were designed and validated in the outpatients setting, as 75% of TEs cases occur in this population, but were not validated in hospitalized patients^8,9^.

Most cancer patients require inpatient care during their disease course, for issues ranging from diagnostic procedures to management of disease- or treatment-related complications. Hospitalization is an independent risk factor for TEs^8^. Moreover, not only does the risk rise upon admission, but it also persists elevated after discharge, with approximately 45% of events occurring in this phase, 75% of which within 45 days^10,11^. To date, international guidelines recommend TP with LMWH in inpatients with active malignancy and acute medical illness or reduced mobility, in the absence of bleeding or other contraindications; without additional risk factors for TEs, the decision whether to provide TP is left to clinicians’ judgement^9^. This uncertainty frequently leads to heterogeneous and sometimes inappropriate prescriptions, with potential drug interactions, preventable side effects and increased health care costs.

Therefore, the definition of a RAM for TEs in hospitalized cancer patients is an unmet need. In detail, given the bleeding risk associated with untailored TP, the identification of patients who might not receive it during in-hospital stay would be clinically useful. Despite its limitations, the KS has demonstrated a high negative predictive value (NPV) in ambulatory cancer patients, thus representing a potential tool also to identify hospitalized patients at negligible TEs risk^6^.

Based on these premises, the main objective of this study was to assess the validity of the KS to predict the risk of TEs during and after hospitalization in a cohort of hospitalized cancer patients. As exploratory objectives, the role of other clinical and biological variables on TEs risk, together with the impact of thrombotic events on patients’ survival, was also evaluated.

## Materials and methods

### Patient population and enrolment criteria

The INDICATE study was a prospective observational study including a consecutive cohort of patients with active cancer hospitalized in the Medical Oncology inpatient clinic at Istituto Nazionale dei Tumori, Milan, Italy. The enrollment criteria consisted in: (1) age ≥ 18 years; (2) histologically proven diagnosis of active, locally advanced or metastatic solid malignancy; (3) hospitalization lasting at least two nights of in-hospital stay; (4) available CT scan confirming the absence of asymptomatic TEs within 3 months before hospitalization; (5) life expectancy > 12 weeks according to clinical judgement at hospitalization. Exclusion criteria were: (1) TEs as reason for hospital admission; (2) history of venous TEs in the past 3 months; (3) ongoing anticoagulant treatment at therapeutic dosage with LMWH or DOACs; given the heterogeneity of TP prescription, and to avoid a selection bias consistently with previous reports^12,13^, patients with ongoing TP at admission, as well as cases where TP was started during in-hospital stay [according to international^9^ and Institutional guidelines or when deemed necessary by the treating clinician (i.e. in case of reduced mobility, sepsis or tumor-related vascular compression)] were included. Similarly, patients receiving antiplatelet drugs including aspirin or clopidogrel were included. Starting from November 2016 [i.e. when institutional electronic medical records (EMRs) were adopted], all consecutive patients were evaluated on the first day of hospitalization and those who fulfilled the eligibility criteria were included. The reason for hospitalization was derived from the main hospitalization-related diagnosis reported in EMRs, that being the main motivation for hospitalization. This led to the inclusion also of patients whose main reason for hospitalization is expected to require less than 2 nights of in-hospital stay (i.e. CVC placement or biopsies), but who experienced procedure-related complications or performed additional investigations resulting in prolonged in-hospital stay.

All patients provided informed consent for use of personal data and were followed up by phone call or visit until death, loss of contact, or time of data lock (31st July 2019). The study was carried out in accordance with the Good Clinical Practice guidelines and the Declaration of Helsinki and was approved by the local institutional review board (IRCCS Central Ethic Committee “Regione Lombardia”—INT 70/20).

### Study outcomes and assessments

Data collection was performed from institutional electronic medical records, including laboratory and imaging results, and review of outside medical records. Clinical and biological patients’ data were collected, including demographics, cancer and treatment characteristics, reason for hospitalization, length of stay and occurrence of fever during in-hospital stay, concomitant anticoagulant or antiplatelet treatments, and blood tests evaluated on the first day of hospitalization. The primary study outcome was the incidence of venous TEs occurring during hospitalization or within 45 days from discharge. Thromboembolic events were defined as (1) deep vein thrombosis (DVT) of the lower or upper limbs, (2) pulmonary embolism (PE), (3) venous visceral or (4) central venous catheter (CVC)-related thrombosis, and needed to be confirmed radiologically (by compressive ultrasound or CT pulmonary angiogram), as per clinical practice. For patients with multiple in-hospital admissions only data concerning the first hospitalization were considered.

### Statistical plan and methods

The primary study objective was to assess the Negative Predictive Value (NPV) of low-grade KS (KS = 0), evaluated at the time of patients’ in-hospital admission, as a tool to predict the risk of TEs in cancer patients during and after (next 45 days) hospitalization. The NPV was defined as the rate of cancer patients with KS = 0 who did not experience TEs. The KS was used according to the published and validated version (0 low, 1–2 intermediate, ≥ 3 high)^5^. In detail, 2 points were assigned to patients with pancreatic and gastric cancers, 1 point to those affected by lung, gynecologic, bladder, testicular cancer or lymphoma, while all other tumor types were considered as low risk and attributed with 0 points.

A single stage design was used. Assuming a 7% of TEs in KS = 0 patients as the unsatisfactory percentage (i.e. H_0_, null hypothesis) and a 3% of TEs as the satisfactory percentage (i.e. H_1_, alternative hypothesis)^12,13^, 149 patients had to be enrolled to detect the favorable NPV with one-sided alpha equal to 0.10 and power equal to 0.80. If seven or less TEs were observed, the NPV would be positively evaluated. Secondary objectives of the study were (a) to evaluate the Positive Predictive Value (PPV) of the KS, as defined as the incidence of TEs among cancer patients with KS ≥ 3 during and after (next 45 days) hospitalization (b) to investigate the impact of TEs on patients’ overall survival (OS); (c) to explore other clinical and biological features, as evaluated on the day of admission, as predictors of TEs risk during and after (next 45 days) hospitalization, in order to develop a new, specific RAM. Therefore, all consecutive eligible patients who fulfilled the inclusion criteria were enrolled irrespectively of the KS, until all the required patients with KS = 0 (estimated to be about 30% of all cases) were included.

Non-parametric descriptive statistics were used to report characteristics of the whole study cohort and according to the occurrence of TEs, with counts and percentages for categorical variables, medians and interquartile range (IQR) for continuous variables, as appropriate. Exact methods were used to estimate the NPV and PPV. Median follow-up was calculated using the reverse Kaplan–Meier method. Survival curves were estimated by the Kaplan–Meier method and compared by log-rank test.

OS was calculated from the first day of hospitalization to death from any cause. Patients alive at the time of data cut-off and analysis were censored at the last date of follow-up. To rule out the likelihood of immortal time bias and to take into account the different lengths of in-hospital stay, a landmark analysis was performed, with OS calculated from the 45th day after discharge to death or last follow up.

As an exploratory analysis, logistic regression was used to identify independent risk factors for TEs, with results summarized as odds ratio (OR) and 95% confidence interval (CI). Predictors of TEs occurrence were considered and screened if evaluable at the first day of in-hospital admission (i.e. excluding those not predictable at the first day of hospitalization, like the length of stay). Variables were then selected via a stepwise backward selection (with a p value threshold of 0.05 to keep variables in the model) and tested in a multivariable model. The final model performance was assessed by examining calibration plots (how close the TEs predicted probabilities were to the actual outcome) and in terms of raw discrimination accuracy (Harrell’s C-index). Internal validation was performed by applying bootstrap re-sampling with 1000 repetitions, both for model calibration and C-index calculation. Comparison between the new score with the KS was performed by means of a decision curve analysis (DCA). Finally, a nomogram was designed to visually describe the effect of predictive factors on TEs risk, as well as a score web calculator. Continuous variables showing marked right skewness were natural logarithm-transformed to avoid a disproportional effect of high values, and missing data were imputed via a random forest model.

The R software (Version 4.0.3) and RStudio software (Version 1.3.1073) [R Foundation for Statistical Computing] were used for statistical analyses.

## Results

### Patients’ characteristics

**Figure 1:**
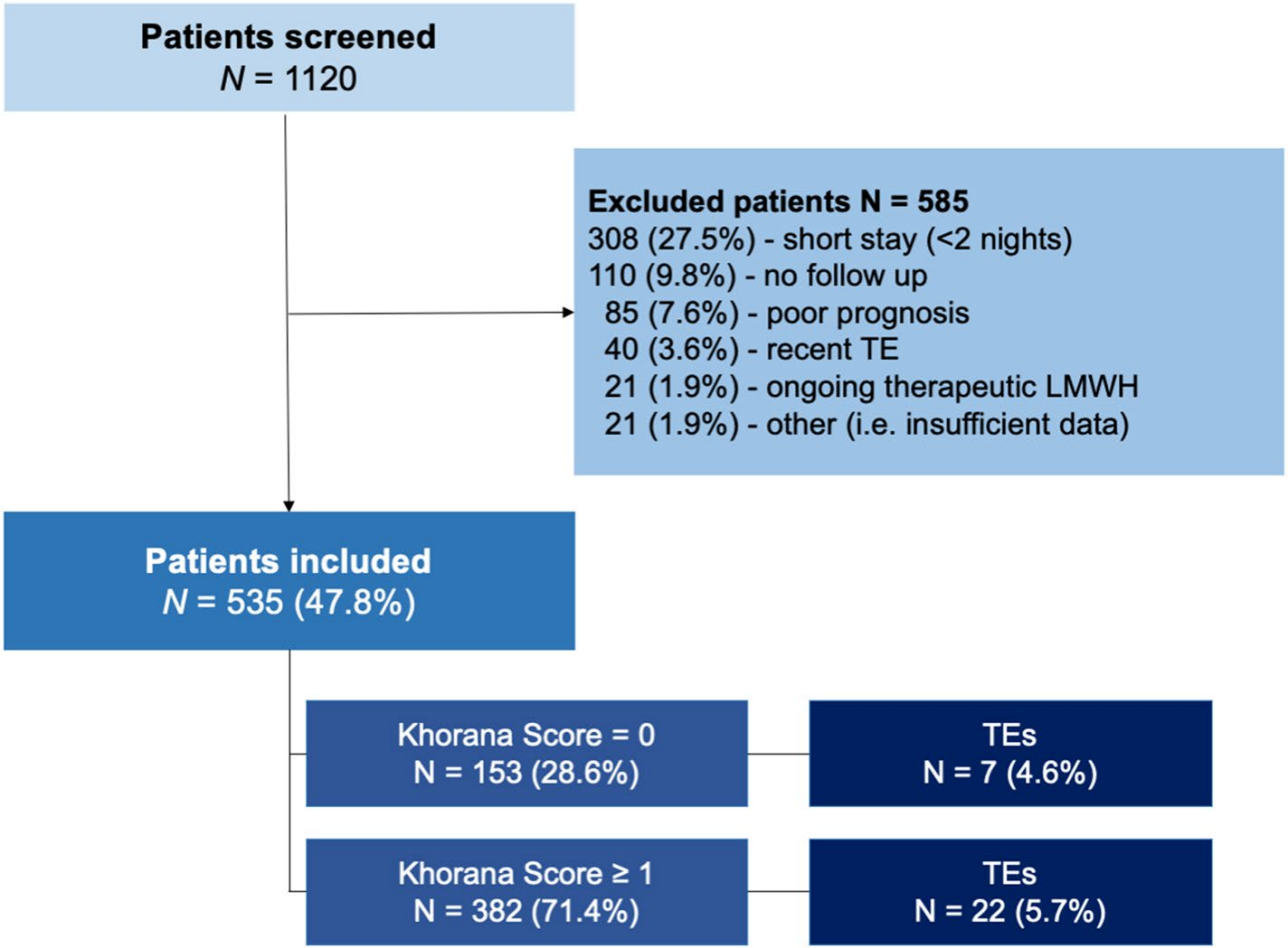
Study flowchart. *TE* thromboembolic event, *LMWH* low molecular weight heparin.

Between November 2016 and May 2019, a total of 1120 consecutive patients were evaluated at their first hospitalization for inclusion. Of these, 585 were excluded due to in-hospital stay shorter than two nights (n = 308, 27.5%), insufficient (< 45 days) follow-up time (n = 110, 9.8%), life expectancy deemed < 12 weeks (n = 85, 7.6%), recent diagnosis of TEs (n = 40, 3.6%), ongoing therapeutic anticoagulant treatment (n = 21, 1.9%) or other causes (n = 21, 1.9%). The study CONSORT diagram is shown in Fig. 1. The final population comprised 535 patients, 153 (28.6%) of whom had a KS = 0. Most patients (55%) had KS = 1 or 2 (28.8% and 26.2%, respectively), while the remaining (16.4%) scored 3 or more points. Patients’ characteristics of the entire study population and according to occurrence of TE events are shown in Table 1.

**Table 1:** Baseline patients’ characteristics in the whole case series and according to the presence or absence of TE events.

Characteristic	Total (N = 535)	Without TE (N = 506)	With TE (N = 29)	p value
Age, median (IQR)	64 (56–72)	64 (56–72)	69 (61–73)	0.292
Gender, male	277 (51.8)	257 (50.8)	20 (69.0)	0.087
BMI, median (IQR)	24 (21–26)	24 (21–26)	23 (22–25)	0.761
≥ 35 kg/m^2^	7 (1.3)	7 (1.4)	–	
**ECOG PS**				0.081
0–1	442 (82.6)	422 (83.4)	20 (69.0)	
≥ 2	93 (17.4)	84 (16.6)	9 (31.0)	
**Smoking status**				0.070
Never	333 (66.5)	316 (66.9)	17 (58.6)	
Former	110 (22.0)	99 (21.0)	11 (38.0)	
Current	58 (11.6)	57 (12.1)	1 (3.4)	
NA	34	34	–	
**Cancer type**				0.909
CUP	9 (1.7)	8 (1.6)	1 (3.4)	
Lung	122 (22.8)	114 (22.5)	8 (27.6)	
Breast	44 (8.2)	43 (8.5)	1 (3.4)	
Colorectal	105 (19.6)	100 (19.8)	5 (17.2)	
Pancreatic	37 (6.9)	35 (6.9)	2 (6.9)	
Gastric and GEJ	75 (14.0)	72 (14.2)	3 (10.3)	
Biliary Tract	29 (5.4)	27 (5.3)	2 (6.9)	
Oesophageal	15 (2.8)	14 (2.8)	1 (3.4)	
Renal	8 (1.5)	8 (1.6)	–	
Melanoma	10 (1.9)	10 (2.0)	–	
Anal	23 (4.3)	22 (4.3)	1 (3.4)	
Prostate	8 (1.5)	7 (1.4)	1 (3.4)	
Bladder	3 (0.6)	3 (0.6)	–	
Other	47 (8.8)	43 (8.5)	4 (13.8)	
**Stage**				0.325
Locally advanced	123 (23.0)	119 (23.5)	4 (13.8)	
Metastatic	412 (77.0)	387 (76.5)	25 (86.2)	
**Number of metastatic sites**				0.248
≤ 2	374 (69.9)	357 (70.6)	17 (58.6)	
> 2	161 (30.1)	149 (29.4)	12 (41.4)	
**Ongoing anticancer treatment platinum-based CT**				0.453
No	277 (51.8)	264 (52.2)	13 (44.8)	
Yes	258 (48.2)	242 (47.8)	16 (55.2)	
**Gemcitabine-based CT**				0.479
No	494 (92.3)	468 (92.5)	26 (89.7)	
Yes	41 (7.7)	38 (7.5)	3 (10.3)	
**Targeted therapy**				0.103
No	458 (85.6)	430 (85.0)	28 (96.6)	
Yes	77 (14.4)	76 (15.0)	1 (3.4)	
**Immune checkpoint inhibitors**				0.425
No	498 (93.3)	473 (93.5)	25 (89.3)	
Yes	36 (6.7)	33 (6.5)	3 (10.7)	
NA	1	–	1	
**Endocrine therapy**				0.282
No	514 (96.3)	488 (96.4)	26 (92.9)	
Yes	20 (3.7)	18 (3.6)	2 (7.1)	
NA	1	–	1	
**Anti-angiogenic therapy**				1.000
No	514 (96.3)	487 (96.2)	27 (96.4)	
Yes	20 (3.7)	19 (3.8)	1 (3.6)	
NA	1	–	1	
**Reason for hospitalization**				**0.016**
Acute respiratory insufficiency	5 (0.9)	5 (1.0)	–	
Biopsy	83 (15.5)	79 (15.6)	4 (13.8)	
Cancer progression/CT toxicity	23 (4.3)	23 (4.5)	–	
CVC placement	19 (3.6)	17 (3.4)	2 (6.9)	
Diarrhea	4 (0.7)	2 (0.4)	2 (6.9)	
Dysphagia	2 (0.4)	1 (0.2)	1 (3.4)	
Fever/acute infection	33 (6.2)	30 (5.9)	3 (10.3)	
Intestinal (sub-)occlusion	6 (1.1)	6 (1.2)	–	
Immune related adverse events	4 (0.7)	4 (0.8)	–	
Malnutrition/cachexia	10 (1.9)	8 (1.6)	2 (6.9)	
Nausea-vomiting	3 (0.6)	3 (0.6)	–	
Obstructive jaundice	2 (0.4)	2 (0.4)	–	
Other	13 (2.4)	11 (2.2)	2 (6.9)	
Pleural effusion	15 (2.8)	15 (3.0)	-	
Refractory pain	11 (2.1)	10 (2.0)	1 (3.4)	
Treatment Administration	302 (56.4)	290 (57.3)	12 (41.4)	
**LOS, days, median (IQR)**	5 (3–8)	5 (3–8)	6 (5–14)	**0.032**
**Fever during hospitalization**	56 (10.5)	51 (10.1)	5 (17.2)	0.361
**Use of antiplatelet agents**	63 (11.8)	59 (11.7)	4 (13.8)	0.960
**Use of LWMH**	56 (10.5)	53 (10.5)	3 (10.3)	1.000
**Khorana score**				0.960
0	153 (28.6)	146 (28.9)	7 (24.1)	
1	154 (28.8)	146 (28.9)	9 (31.0)	
2	140 (26.2)	131 (25.8)	8 (27.6)	
≥ 3	88 (16.4)	83 (16.4)	5 (17.2)	
**Vascular compression**	63 (12.1)	51 (10.3)	12 (44.4)	**< .001**
NA	13	11	2	
**Previous TEs**	29 (5.4)	25 (5.0)	4 (14.3)	0.058
NA	2	1	1	

Data are presented as n (%) except where otherwise noted. The p value of the χ2 test, Fisher’s exact test o WMW test assessing the association between each characteristic and the occurrence of TE events is indicated in the right column of the table. The p value of the test is indicated in bold numbers when statistically significant.

*BMI* body mass index, *CT* chemotherapy, *CUP* cancer of unknown primary, *CVC* central venous catheter, *ECOG PS* Eastern Cooperative Oncology Group Performance Status, *GEJ* gastro-esophageal junction, *IQR* interquartile range, *LMWH* low molecular weight heparin, *LOS* length of stay, *NA* not available, *TE* thromboembolic event.

Overall, 29 (5.4%) patients developed a thromboembolic event during hospitalization or within 45 days from discharge, including 14 patients with PE, 6 with DVT, 2 with both PE and DVT, 4 with visceral thrombosis and 3 with CVC-related thrombosis; of these, 41.4% and 58.6% occurred during and after hospitalization, respectively. Among the 16 cases of PE, 8 cases were deemed as minor (asymptomatic, segmental), 3 cases as moderate (symptomatic, segmental) and 5 cases as severe PE (heavily symptomatic and/or bilateral). Detailed characteristics of TEs are reported in Table 2.

**Table 2:** Detailed characteristics of thromboembolic events.

	Total (N = 29)
**Time since hospitalization (days)**	
Median (range)	13 (1–52)
**Timing**	
During hospitalization	12 (41.4)
Post hospitalization	17 (58.6)
**Type of TE**	
Pulmonary embolism	14 (48.3)
Asymptomatic, segmental	7 (24.1)
Symptomatic, segmental	3 (10.3)
Heavily symptomatic and/or bilateral	4 (13.8)
Deep venous thrombosis + Pulmonary embolism	2 (6.9)
Asymptomatic, segmental	1 (3.4)
Heavily symptomatic and/or bilateral	1 (3.4)
Deep venous thrombosis	6 (20.7)
Visceral thrombosis	4 (13.8)
CVC-related thrombosis	3 (10.3)
**Symptoms**	10 (34.5)
**Bleeding**	
Major	2 (6.9)
Minor	1 (3.4)
**Death due to TE**	1 (3.4)

*CVC* central venous catheter, *TE* thromboembolic event.

Demographic and disease characteristics were similar between patients who developed TEs and those who did not (see Table 1). The reason for in-hospital admission had a markedly varying distribution, with higher TE incidence among patients admitted for CVC placement, diarrhea, dysphagia, fever/acute infection, malnutrition/cachexia, and refractory pain. Moreover, patients who experienced TEs had a longer length of in-hospital stay (p = 0.03). No significant differences emerged in terms of tumor types and ongoing antitumor treatments.

Of note, the use of prophylactic LMWH was not associated with a lower rate of TEs (p = 1.00). To further elucidate the role of TP in our cohort, we evaluated patients’ characteristics according to use of LMWH prophylaxis (Supplementary Table 1). As expected, patients treated with LMWH had overall poorer clinical conditions, i.e. worse ECOG PS, higher tumor burden (in terms of number of metastatic disease sites and presence of tumor-related vascular compression), longer in-hospital stay and higher rates of fever. Notably, no patients with low Khorana Score (KS = 0) received TP.

Baseline blood parameters are provided in Supplementary Table 2, showing that, patients experiencing TEs had lower median levels of hemoglobin (p = 0.009) and albumin (p =  < 0.001), and higher levels of C-reactive protein (CRP, p = 0.003) and LDH (p =  < 0.001).

### Predictive value of the Khorana score on TEs risk

The study met its primary endpoint: out of 153 patients who had a low KS, 7 (4.6%) had a thromboembolic event, resulting in a NPV of KS = 0 of 95.4% (One-sided exact 90% Lower Confidence Limit: 93.2%, one-sided p-value: 0.084; exact 95% CI 90.8–98.1%). A high (≥ 3) Khorana score was not significantly associated with an increased risk of TEs, resulting in a PPV of 5.7% (95% CI 1.9–12.8%): the incidence of thrombosis was 5.8% (9/154) in patients with KS = 1, 5.7% (8/140) in patients with KS = 2 and 5.7% (5/88) in those with KS ≥ 3.

### Development of TEs risk nomogram

Results of univariable and multivariable logistic regression analysis testing the effect of specific covariates on the risk of TEs are shown in Supplementary Table 3. LDH and CRP levels showed a markedly right skewed distribution and therefore were natural logarithm-transformed to avoid a disproportional effect of high values. In a stepwise multivariable model, albumin (OR 0.34; 95% CI 0.17–0.70, p = 0.003) and log-transformed LDH levels (OR 1.90; 95% CI 1.07–3.24, p = 0.022), together with presence of tumor-related vascular compression (OR 5.35; 95% CI 2.30–12.10, p < 0.001) resulted as significantly associated with risk of TEs. Estimated probabilities of developing TEs could thus be calculated according to the following formula: P = 1/{1 + exp [− (“TE score”)]}, where “TE score” is derived from β-coefficients of the multivariable model, as follows: [− 3.1497 + (− 1.0709 × Albumin) + (0.6464 × log(LDH)) + (1.6745 × Vascular compression)]. An online calculator for the score can be found at https://federico-nichetti.shinyapps.io/indicate_webcalc/.

Based on Harrel’s C-index, the discriminatory performance of this model was 0.78 (95% CI 0.69–0.87, see Supplementary Fig. 1). Internal validation by bootstrapping resulted in an adjusted C-index of 0.77, representing hardly any optimism (i.e. 0.0094). The model’s calibration plot is shown in Supplementary Fig. 2: most TEs predicted probabilities were concentrated in low ranges, where a good agreement with observed proportions was documented. A DCA showed how the standardized net benefit of the new score surpasses the standardized net benefit of the KS for quite all probability thresholds. The new score is also superior to the two extreme strategies of treating all the patients and of treating none of the patients with TP (see Supplementary Fig. 3).

**Figure 2:**
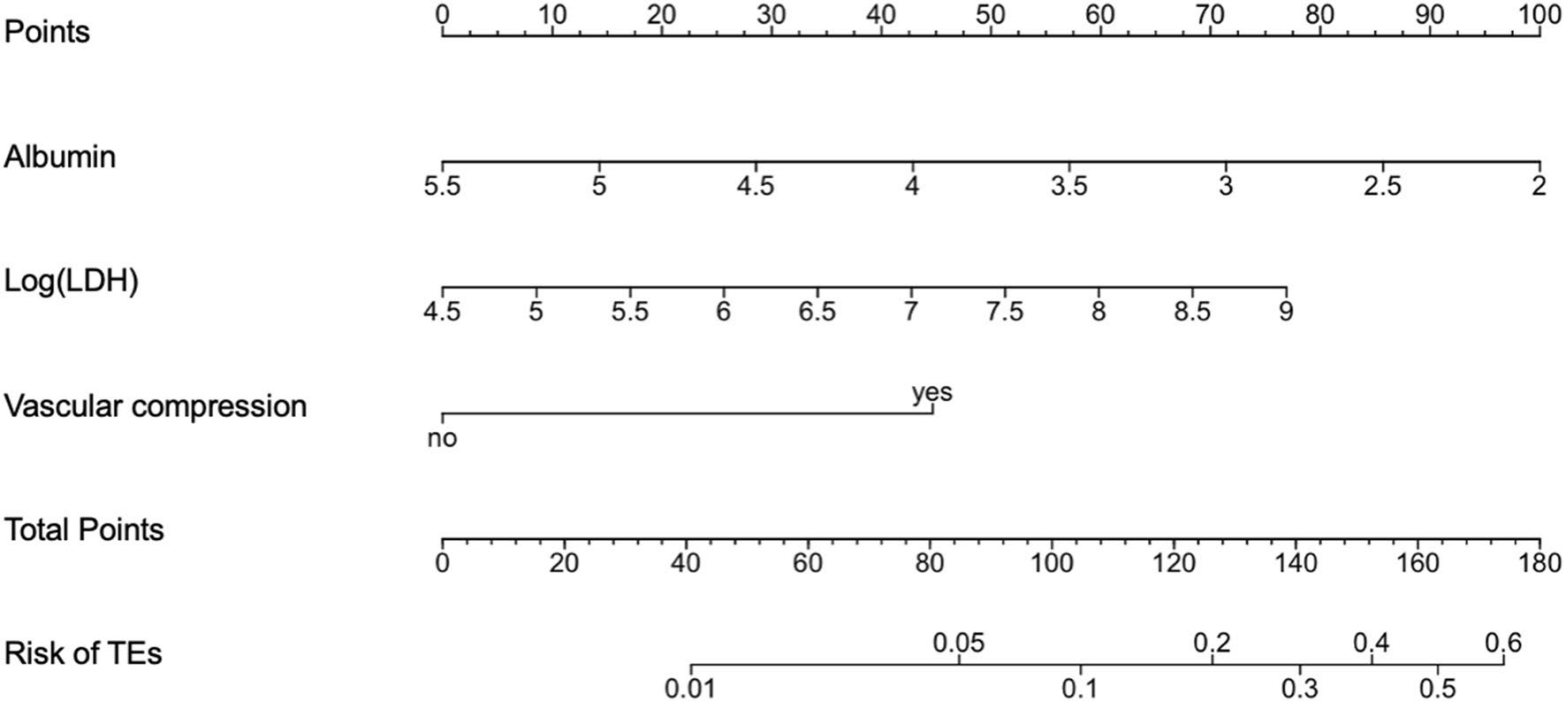
Nomogram predicting the probability of developing a TE during hospitalization and in the next 45 days after discharge. *Log(LDH)* natural logarithm lactate dehydrogenase, *TEs* thromboembolic events.

Based on these results, and to make TEs prediction easier for clinical practice, we developed a nomogram scoring system to predict the risk of TEs in hospitalized cancer patients. The nomogram is shown in Fig. 2 and predicts the risk of TEs during and in the 45 days after hospitalization of at least two nights in cancer patients. In this nomogram, presence of vascular compression, together with lower values of albumin and higher values of LDH results in higher TEs risk.

To rule out a potential bias linked to use of LMWH-based TP, we performed a supplementary analysis excluding patients treated with LMWH during the study observation time. Supplementary Table 4 reports univariable and multivariable logistic regression analysis in this subset: as previously, backward variable selection resulted in the same predictors of TEs.

### Impact of TEs on patients’ survival

**Figure 3:**
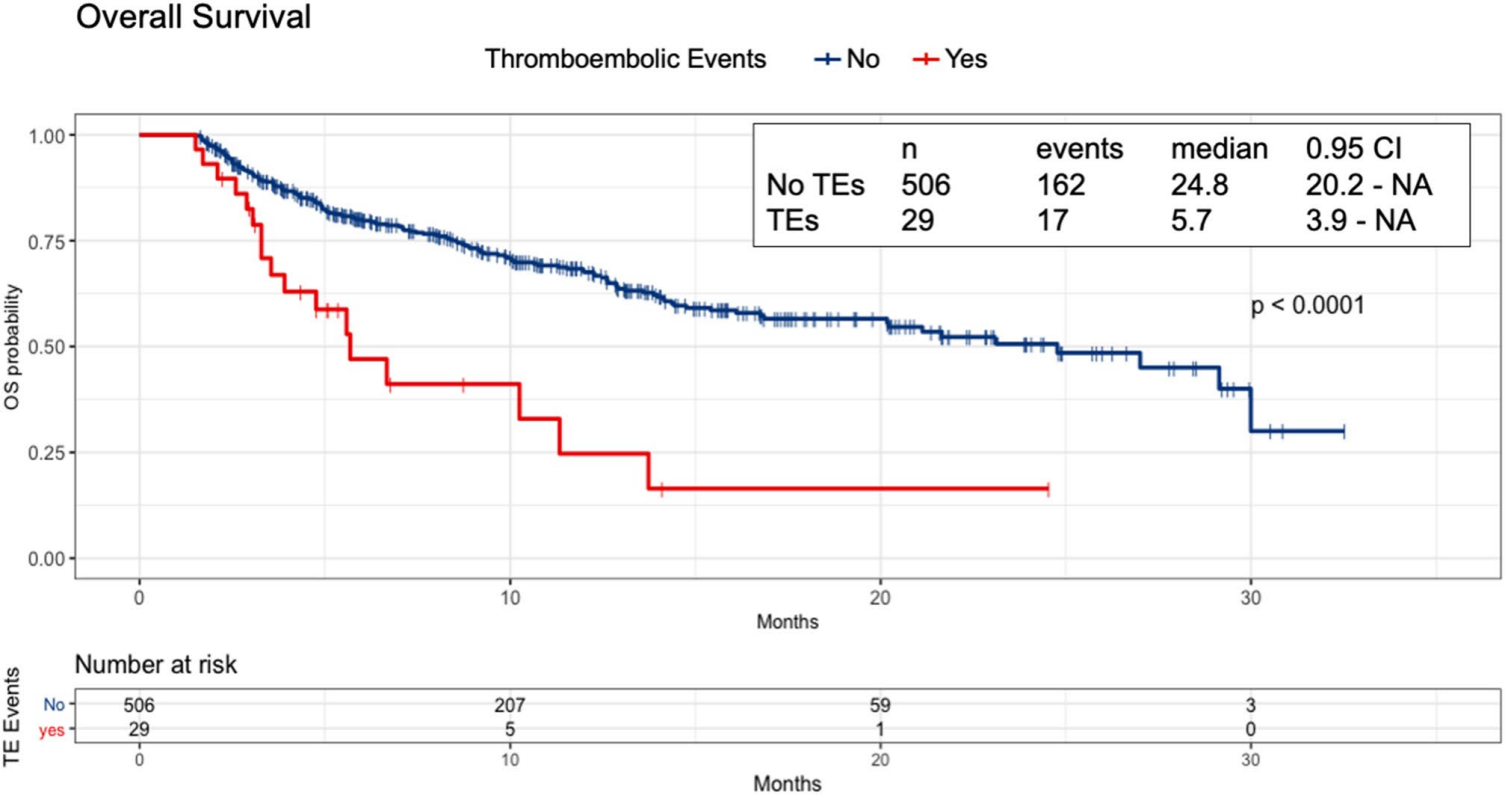
Kaplan Meier curves of Overall Survival according to the occurrence of thromboembolic events during or after hospitalization. *NA* not available (not reached), *OS* Overall survival, *TEs* thromboembolic events.

The median duration of follow-up was 11.5 months (95% CI 10.4–12.8). An exploratory OS analysis, based on 179 (33.5%) deaths among the 535 patients, was performed. In patients who had TEs, the median OS time was 5.7 months (95% CI 3.9–NA) versus 24.8 months (95% CI 20.2–NA) in the group who did not develop TEs [hazard ratio (HR) 2.98, 95% CI 1.08–4.94, p ≤ 0.001]. The Kaplan–Meier curves of OS in patients with or without TEs are displayed in Fig. 3. Seven patients died during the study observation window (i.e. within 45 days from discharge). At a landmark analysis starting from the 45th day after discharge, TEs remained significantly associated with poorer OS [median, 5.1 months (95% CI 3.2–NA) versus 23.1 months (95% CI 18.4–NA), HR 2.83, 95% CI 1.66–4.83, p ≤ 0.001, see Supplementary Fig. 4].

## Discussion

Hospitalized cancer patients have a high risk of thrombotic complications, resulting in increased morbidity and mortality. At the same time, these patients are also at increased risk for bleeding, so that the prevention of one adverse event may result in an unjustified higher risk of the other^10^. Therefore, it is a matter of primary importance to define the best strategy to prevent thrombosis without causing bleeding as a side effect. According to current international guidelines, hospitalized patients who have active malignancy without additional risk factors do not have a strict recommendation for TP, thus leaving a gray area and resulting in frequent but non-targeted prescription of LMWH.

The INDICATE study was designed with the aim of shrinking this gap of knowledge by providing real-world data on TEs risk assessment in hospitalized patients with solid tumors. We observed a 5.4% rate of TEs, consistent with the pre-existing data reporting an overall TEs incidence ranging around 4.1% to 8.4%^10,12–15^. Our study met its primary endpoint, as a low-risk KS (KS = 0) was associated with a NPV for TEs of > 95%.

Initial, retrospective series investigated the role of this score for TEs risk assessment in the hospitalized cancer population^12,13^. In these studies, the rate of TEs among patients with low-risk KS was 2.5% and 1.4%, respectively, supporting our hypothesis that the KS may serve as a tool to identify hospitalized cancer patients that may avoid unnecessary prophylaxis. Both studies showed that patients with KS ≥ 2 had a significantly higher TEs risk (OR 1.8 and 2.3 in^12^ and^13^, respectively) compared to those with 0–1. This result was not confirmed in our series, with multiple potential explanations. First, previous reports showed that the KS has modest applicability in the European population, also because of a lower rate of severely obese individuals^16–18^. In addition, our study focused also on post-discharge TEs, which were not considered in the two previous series^12,13^. Secondly, the low absolute event occurrence (n = 29) and the inclusion of subjects on TP may have masked the PPV of high KS. Finally, our study was conducted according to a specific statistical plan based on the NPV of the score, so that demonstrating an overall calibration of the KS would be problematic. The choice of NPV as main endpoint was motivated by two reasons: first, the NPV is reported as the main strength of the KS in the outpatient setting. Secondly, as we aimed to check absolute limits of the negative predictive error (i.e. no more than 7% of false negative diagnoses was allowed), we preferred to evaluate the NPV in absolute terms (i.e. percentage of patients with KS = 0 not experiencing any TE) instead of a relative measure such as the negative likelihood ratio [i.e. the probability that a patient experiencing a TE had KS = 0 (false negative) divided by the probability that a patient without TE had KS = 0 (true negative)]. Given these results, the use of the KS for hospitalization-related TEs risk assessment is still debatable.

Other than the KS, no other RAMs have been validated for the thrombotic risk of hospitalized cancer patients so far. To this aim, and to provide new insights on the biological mechanisms underlying the development of thrombosis in this population, we evaluated the association between clinical and biological variables and TEs risk. Intuitively, patients with tumor-related vascular compression had higher rates of TEs; indeed, vascular compression has been previously included in the ONKOTEV outpatients’ RAM^19^. Intriguingly, patients who experienced TEs had lower median albumin and higher LDH and CRP serum levels compared to those who did not^20^. The association between albumin levels and thrombosis occurrence in patients with cancer has been previously reported. Shah and colleagues showed a 39% venous TE risk reduction among patients with albumin levels ≥ 4 g/dL (HR 0.61)^21^. More recently, a large cohort study conducted on > 1000 cancer patients in the framework of the Vienna CAT study specifically evaluated the association between low serum albumin and increased TE risk. In this study, patients with albumin levels below 4.42 g/dL had a 2.2-fold increased risk of TE, compared to those with serum albumin above this cut-off^22^. Concerning LDH, high serum levels have been shown as an independent risk factor for TEs in patients with diffuse large b-cell lymphoma or germ cell tumors undergoing platinum-based chemotherapy^23–26^. Given our data, we hypothesize that low albumin and high LDH and CRP serve as markers of cancer-related systemic inflammation and cachexia, resulting also in a greater hypercoagulable state in these patients. In this light, TEs occur as manifestations of disease progression in patients with advanced and uncontrolled tumors^27^. This hypothesis is further supported by the evidence that patients experiencing TEs had significantly poorer survival. Of note, the Glasgow prognostic score, an outcome prediction model for cancer patients, is based on CRP and albumin levels^28^. In this light, TEs might represent signs of advanced disease rather than direct causes of poorer survival^29^. Clearly, given that the OS analysis could be biased by the fact that patients were hospitalized at different times in their oncological history (i.e. at diagnosis *vs* at a very advanced phase), the evidence of reduced survival in patients with TEs just serves as proof of concept for our hypothesis.

Based on these data, we developed a nomogram scoring system that can be used to facilitate the decision process concerning the prescription of TP in this population. In detail, this nomogram estimates up to 60% of risk of developing TEs, and easily identifies the majority of patients for which the TEs risk related to hospitalization is less than 10%. With these characteristics, while risk thresholds for considering TP are subjective from both the clinicians’ and patients’ perspective, the nomogram might help to select cases in whom the increased risk of bleeding due to TP would outweigh the benefits. In particular, such a nomogram could be especially useful to evaluate TP in cancer patients hospitalized without active medical illness or with TP contraindications, where no clear guidelines indications exist. However, it must be explicit that, despite our internal validation, this new RAM was developed on a very limited number of TEs and in a monocentric cohort, thus clearly requiring an external assessment in a larger patient population to be considered robust. Nonetheless, this new score serves as a proof of concept, and will hopefully lead to more extensive evaluation of these blood parameters in the near future.

Our study has other clear limitations. First, the monocentric nature of the observation, the relatively small sample size (given the rarity of the event of interest), together with the lack of a scheduled assessment for TEs development, might have affected our results. In particular, a number of asymptomatic events might have been missed. Concerning the cohort selection, cases hospitalized for only one night were excluded, as hospitalization was unlikely to provide an additional risk factor for TEs in these patients; however, this limits the applicability of a RAM in real life, as clinicians need to estimate the length of in-hospital stay at admission to use the model for TP decisions. Moreover, our series did not include some types of cancers, like sarcomas, head & neck cancers or gynecological tumors. Additionally, the inclusion of patients on TP before enrolment could have prevented a number of events in high-risk patients. Indeed, we did not observe an evident benefit from TP with LMWH, as in previous series^12,13,29^. However, in our population TP was administered with different dosing and duration schedules, thus limiting the reliability of the deriving evidence, and highlighting the limited application of current guidelines. Nonetheless, a recent phase 2 study by Zwicker and colleagues evaluated the feasibility of TP with weight-based LMWH, showing its efficacy in preventing TEs in hospitalized cancer patients^30^. Moreover, we performed a subset analysis excluding patients on TP, showing overlapping results with our previous data and thus adding robustness to our findings.

## Conclusions

In conclusion, our study demonstrated that a low KS may serve as a negative predictor of thrombosis in hospitalized cancer patients, though the overall performance of the KS was poor. Therefore, we developed a new nomogram for TEs prediction, based on clinical and laboratory features evaluated at in-hospital admission. In our opinion, this nomogram can be a useful tool in daily clinical practice, especially in cases where there is no clear indication for TP according to current guidelines, and deserves further assessment in external cohorts. Based on these RAMs, we think future trials could evaluate the safety of avoiding TP in patients we identified as having a low-risk hospitalization-related TEs.

## Supplementary Information

Supplementary Information.
