# Supplementary material for: Risk assessment of thromboembolic events in hospitalized cancer patients
Source: Sci Rep. 2021 Sep 14;11:18200. doi: 10.1038/s41598-021-97659-9 (PMC8440577; doi:10.1038/s41598-021-97659-9)
Supplement: Supplementary file 1 — Supplementary Information. [file 41598_2021_97659_MOESM1_ESM.docx]

**Supplementary Tables and Figures**

**Supplementary Table 1.** Patient’s characteristics according to the use of LMWH-based TP.

| **Characteristic** | **Total (N=535)** | **no LMWH (N=479)** | **LMWH (N=56)** | **p value** |
| --- | --- | --- | --- | --- |
| **TE** | 29 (5.4) | 26 (5.4) | 3 (5.4) | 1.000 |
| **Age**, median (IQR) | 64 (56-72) | 63 (55-72) | 68 (61 -71) | **0.048** |
| **Gender**, male | 277 (51.8) | 246 (51.4) | 31 (55.4) | 0.672 |
| **BMI,** median (IQR)  ≥35 kg/m^2^ | 24 (21–26)  7 (1.3) | 24 (21-26)  7 (1.5) | 24 (22-26)  - | 0.883  1.000 |
| **ECOG PS**     0-1     ≥2 | 442 (82.6)  93 (17.4) | 409 (85.4)  70 (14.6) | 33 (58.9)  23 (41.1) | **<.001** |
| **Smoking status**     Never     Former     Current     NA | 333 (66.5)  110 (22.0)  58 (11.5)  34 | 306 (67.7)  93 (20.6)  53 (11.7)  27 | 27 (55.1)  17 (34.7)  5 (10.2)  7 | 0.082 |
| **Cancer type**     CUP   Lung     Breast     Colorectal     Pancreatic     Gastric and GEJ     Biliary Tract     Oesophageal     Renal     Melanoma     Anal     Prostate     Bladder     Other | 9 (1.7)  122 (22.8)  44 (8.2)  105 (19.6)  37 (6.9)  75 (14.0)  29 (5.4)  15 (2.8)  8 (1.5)  10 (1.9)  23 (4.3)  8 (1.5)  3 (0.6)  47 (8.8) | 9 (1.9)  96 (20.0)  38 (7.9)  104 (21.7)  33 (6.9)  70 (14.6)  29 (6.1)  15 (3.1)  7 (1.5)  8 (1.7)  22 (4.6)  7 (1.5)  3 (0.6)  38 (7.9) | -  26 (46.4)  6 (10.7)  1 (1.8)  4 (7.1)  5 (8.9)  -  -  1 (1.8)  2 (3.6)  1 (1.8)  1 (1.8)  -  9 (16.1) | **<.001** |
| **Stage**     Locally advanced     Metastatic | 123 (23.0)  412 (77.0) | 112 (23.4)  367 (76.6) | 11 (19.6)  45 (80.4) | 0.616 |
| **Number of metastatic sites**  ≤2     >2 | 374 (69.9)  161 (30.1) | 341 (71.2)  138 (28.8) | 33 (58.9)  23 (41.1) | 0.059 |
| **Ongoing anticancer treatment**  **Platinum-based CT**     no     yes | 277 (51.8)  258 (48.2) | 246 (51.4)  233 (48.6) | 31 (55.4)  25 (44.6) | 0.571 |
| **Gemcitabine-based CT**     no     yes | 494 (92.3)  41 (7.7) | 445 (92.9)  34 (7.1) | 49 (87.5)  7 (12.5) | 0.150 |
| **Targeted therapy**     no     yes | 458 (85.6)  77 (14.4) | 411 (85.8)  68 (14.2) | 47 (83.9)  9 (16.1) | 0.705 |
| **Immune checkpoint inhibitors**     no     yes  NA | 498 (93.3)  36 (6.7)  1 | 448 (93.7)  30 (6.3)  1 | 50 (89.3)  6 (10.7)  - | 0.210 |
| **Endocrine therapy**     no     yes  NA | 514 (96.3)  20 (3.7)  1 | 460 (96.2)  18 (3.8)  1 | 54 (96.4)  2 (3.6)  - | 1.000 |
| **Anti-angiogenic therapy**     no     yes  NA | 514 (96.3)  20 (3.7)  1 |  |  | 1.000 |
|  |  | 460 (96.2)  18 (3.8)  1 | 54 (96.4)  2 (3.6)  0 |  |
| **Reason for hospitalization**     Acute Respiratory Insufficiency     Biopsy     Cancer Progression/CT Toxicity     CVC placement     Diarrhea     Dysphagia     Fever / Acute Infection     Intestinal (sub-)occlusion     Immune related adverse events     Malnutrition / Cachexia     Nausea-Vomiting     Obstructive jaundice     Other     Pleural Effusion     Refractory Pain     Treatment Administration | 5 (0.9)  83 (15.5)  23 (4.3)  19 (3.6)  4 (0.7)  2 (0.4)  33 (6.2)  6 (1.1)  4 (0.7)  10 (1.9)  3 (0.6)  2 (0.4)  13 (2.4)  15 (2.8)  11 (2.1)  302 (56.4) | 2 (0.4)  76 (15.9)  20 (4.2)  16 (3.3)  4 (0.8)  2 (0.4)  25 (5.2)  6 (1.3)  3 (0.6)  9 (1.9)  3 (0.6)  2 (0.4)  9 (1.9)  11 (2.3)  10 (2.1)  281 (58.7) | 3 (5.4)  7 (12.5)  3 (5.4)  3 (5.4)  -  -  8 (14.3)  -  1 (1.8)  1 (1.8)  -  -  4 (7.1)  4 (7.1)  1 (1.8)  21 (37.5) | **0.004** |
| **LOS,** median (IQR) | 5 (3-8) | 5 (3-8) | 8 (4-15) | **<.001** |
| **Fever during hospitalization** | 56 (10.5) | 39 (8.1) | 17 (30.4) | **<.001** |
| **Use of antiplatelet agents** | 63 (11.8) | 60 (12.5) | 3 (5.4) | 0.129 |
| **Khorana Score**     0     1     2     ≥3 | 153 (28.6)  155 (29.0)  139 (26.0)  88 (16.4) | 153 (31.9)  131 (27.3)  122 (25.5)  73 (15.2) | -  24 (42.9)  17 (30.4)  15 (26.8) | **<.001** |
| **Vascular compression**     NA | 63 (12.1)  13 | 51 (10.9)  12 | 12 (21.8)  1 | **0.019** |
| **Previous TEs**     NA | 29 (5.4)  2 | 23 (4.8)  1 | 6 (10.9)  1 | 0.059 |
| Data are presented as n (**%**) except where otherwise noted. The p value of the χ2 test, Fisher’s exact test o WMW test assessing the association between each characteristic and the occurrence of TE events is indicated in the right column of the table. The p value of the test is indicated in bold numbers when statistically significant.  Abbreviations: BMI: body mass index; CT: chemotherapy; CUP: cancer of unknown primary; CVC: central venous catheter; ECOG PS: Eastern Cooperative Oncology Group Performance Status; GEJ: gastro-esophageal junction; IQR: interquartile range; LMWH: low molecular weight heparin; LOS: length of stay; NA: not available; TE: thromboembolic event. | | | | |

**Supplementary Table 2.** Baseline laboratory values in the whole case series and according to the occurrence of thromboembolic events.

|  | Total  (N = 535) | Without TE  (N = 506) | With TE  (N = 29) | p value |
| --- | --- | --- | --- | --- |
| WBCs [/μL]  median (IQR)    >11000 | 7620 (5760 - 10220)  105 (19.6) | 7655 (5762 - 10230)  100 (19.8) | 7250 (5760 - 9270)  5 (17.2) | 0.846  0.740 |
| Hemoglobin [g/dl]  median (IQR)    <10 | 12.3 (11.0 - 13.4)  67 (12.5) | 12.3 (11.1 - 13.4)  61 (12.1) | 11.5 (10.2 - 12.7)  6 (20.7) | **0.009**  0.281 |
| Platelets [*10^9^/L]  median (IQR)    ≥350 | 280 (215 - 366)  152 (28.4) | 280 (217 - 366)  145 (28.7) | 276 (182 - 309)  7 (24.1) | 0.316  0.754 |
| Neutrophils [/μL]  median (IQR) | 5200 (3800 - 7650) | 5200 (3800 - 7600) | 5200 (4000 - 8000) | 0.730 |
| Monocytes [/μL]  median (IQR) | 400 (300 - 600) | 400 (300 - 600) | 500 (300 - 600) | 0.771 |
| Albumin [g/dl]  median (IQR)  NA | 4.0 (3.6 - 4.3)  23 | 4.0 (3.7 - 4.3)  23 | 3.7 (3.2 - 4.0)  - | **<.001** |
| CRP [mg/L]  median (IQR)  NA | 11 (2 - 46)  64 | 11 (2 - 44)  60 | 42 (19 - 117)  4 | **0.003** |
| Fibrinogen [mg/dl]  median (IQR)  NA | 423 (343 - 552)  62 | 424 (344 - 552)  55 | 416 (315 - 540)  7 | 0.557 |
| LDH [U/L]    median (IQR)  NA | 351 (282 - 483)  30 | 346 (281 - 473)  30 | 485 (341 - 574)  - | **<.001** |
| All blood tests were performed at Istituto Nazionale dei Tumori of Milan. Cut-offs for hemoglobin, white blood cells and platelets counts were chosen according to the Khorana Score. The p value is indicated in the right column of the table and in bold numbers when statistically significant.  Abbreviations: CRP: C-reactive protein; IQR: interquartile range; LDH: lactate dehydrogenase; NA: not available; TE: thromboembolic event; WBCs: white blood cells. | | | | |

**Supplementary Table 3.** Univariable and multivariable binary logistic regression analyses of association between clinical and biological variables and TEs.

| Characteristic |  | Univariable  OR (95 CI, p) | Multivariable  OR (95 CI, p) |
| --- | --- | --- | --- |
| Age | Continuous | 1.02 (0.99-1.05, p=0.299) | - |
| Gender | Male vs female | 2.15 (0.99-5.06, p=0.062) | - |
| BMI | Continuous | 0.98 (0.88-1.07, p=0.640) |  |
| ECOG PS | ≥2 vs 1 | 2.26 (0.95-5.00, p=0.051) | - |
| Smoking status | Never  Former  Current | -  2.22 (0.98-4.85, p=0.048)  0.35 (0.02-1.76, p=0.315) | -  -  - |
| Stage | Locally advanced vs metastatic | 1.92 (0.73-6.62, p=0.234) | - |
| Number of metastatic sites | ≤2 vs >2 | 1.69 (0.77-3.60, p=0.177) | - |
| Platinum-based CT | Yes vs no | 1.34 (0.63-2.90, p=0.443) | - |
| Gemcitabine-based CT | Yes vs no | 1.42 (0.33-4.28, p=0.579) |  |
| Targeted therapy | Yes vs no | 0.20 (0.01-0.97, p=0.119) | - |
| Immune checkpoint inhibitors | Yes vs no | 1.65 (0.38-5.02, p=0.429) | - |
| Endocrine therapy | Yes vs no | 2.01 (0.31-7.46, p=0.366) | - |
| Antiangiogenic therapy | Yes vs no | 0.92 (0.05-4.67, p=0.933) | - |
| Use of antiplatelet agents | Yes vs no | 1.21 (0.35-3.26, p=0.729) | - |
| Use of LMWH | Yes vs no | 0.99 (0.23-2.92, p=0.982) | - |
| Khorana Score | 0  1  2  ≥3 | -  1.29 (0.47-3.68, p=0.627)  1.27 (0.45-3.72, p=0.649)  1.26 (0.36-4.06, p=0.704) | -  -  -  - |
| Vascular compression | Yes vs no | 6.30 (2.79-13.85, p<.001) | 5.35 (2.30-12.10, p<.001) |
| Previous TEs | Yes vs no | 3.08 (0.86-8.71, p=0.051) | - |
| WBC [/μL] | Continuous | 1.00 (1.00-1.00, p=0.776) | - |
| Hemoglobin [g/dl] | Continuous  < 10 vs ≥10 | 0.75 (0.62-0.90, p=0.002)  0.53 (0.22-1.47, p=0.179) | -  - |
| Platelets [*10^9^/L] | Continuous | 1.00 (1.00-1.00, p=0.805) | - |
| Neutrophils [/μL] | Continuous | 1.00 (1.00-1.00, p=0.774) | - |
| Monocytes [/μL] | Continuous | 1.00 (1.00-1.00, p=0.990) | - |
| Albumin [g/dl] | Continuous | 0.29 (0.15-0.57, p<.001) | 0.34 (0.17-0.70, p=0.003) |
| Log(CRP) | Continuous | 1.54 (1.19-2.03, p=0.002) | - |
| Fibrinogen [mg/dl] | Continuous | 1.00 (1.00-1.00, p=0.853) | - |
| Log(LDH) | Continuous | 2.29 (1.33-3.79, p=0.002) | 1.90 (1.07-3.24, p=0.022) |
| Abbreviations: BMI: body mass index; CRP: C-reactive protein; CT: chemotherapy; LDH: lactate dehydrogenase; ECOG PS: Eastern Cooperative Oncology Group Performance Status; LMWH: low molecular weight heparin; NA: not available; TE: thromboembolic event; WBC: white blood cells. | | | |

**Supplementary Table 4.** Univariate and multivariable binary logistic regression analyses of association between clinical and biological variables and TEs in patients not treated with low-molecular-weight heparin thromboprophylaxis.

| Characteristic |  | Univariable  OR (95 CI, p) | Multivariable  OR (95 CI, p) |
| --- | --- | --- | --- |
| Age | Continuous | 1.03 (1.00-1.07, p=0.113) | - |
| Gender | Male vs Female | 1.85 (0.82-4.42, p=0.147) | - |
| BMI | Continuous | 0.99 (0.89-1.09, p=0.867) | - |
| ECOG PS | ≥2 vs 0-1 | 2.80 (1.11-6.53, p=0.021) | - |
| Smoking status | Never  Former  Current | -  2.48 (1.04-5.66, p=0.033)  0.41 (0.02-2.06, p=0.387) | -  -  - |
| Stage | Locally advanced vs metastatic | 1.72 (0.64-5.98, p=0.327) | - |
| Number of metastatic sites | >2 vs ≤2 | 1.88 (0.82-4.19, p=0.123) | - |
| Platinum-based CT | Yes vs no | 1.25 (0.56-2.80, p=0.586) | - |
| Targeted-therapy | Yes vs no | 0.23 (0.01-1.12, p=0.153) | - |
| Immune checkpoint inhibitors | Yes vs no | 2.06 (0.47-6.40, p=0.263) | - |
| Endocrine therapy | Yes vs no | 2.28 (0.35-8.64, p=0.291) | - |
| Antiangiogenic therapy | Yes vs no | 1.03 (0.06-5.32, p=0.981) | - |
| Antiplatelet Therapy | Yes vs no | 1.29 (0.37-3.52, p=0.651) | - |
| Khorana Score | 0  1  2  ≥3 | -  1.36 (0.47-3.97, p=0.566)  1.27 (0.42-3.81, p=0.664)  1.21 (0.31-4.14, p=0.768) | -  -  -  - |
| Vascular compression | Yes vs no | 6.28 (2.60-14.58, p<.001) | 5.91 (2.33-14.57, p<.001) |
| Previous TEs | Yes vs no | 2.82 (0.63-9.02, p=0.113) | - |
| WBC [/μL] | Continuous | 1.00 (1.00-1.00, p=0.606) | - |
| Hemoglobin [g/dl] | Continuous  < 10 vs ≥10 | 0.74 (0.61-0.90, p=0.002)  0.40 (0.16-1.15, p=0.064) | -  - |
| Platelets [*10^9^/L] | Continuous | 1.00 (1.00-1.00, p=0.895) | - |
| Neutrophils [/μL] | Continuous | 1.00 (1.00-1.00, p=0.902) | - |
| Monocytes [/μL] | Continuous | 1.00 (1.00-1.00, p=0.946) | - |
| Albumin [g/dl] | Continuous | 0.21 (0.10-0.44, p<.001) | 0.22 (0.10-0.49, p<.001) |
| Log(CRP) | Continuous | 1.54 (1.17-2.07, p=0.003) | - |
| Fibrinogen [mg/dl] | Continuous | 1.00 (1.00-1.00, p=0.488) | - |
| Log(LDH) | Continuous | 2.43 (1.38-4.12, p=0.001) | 1.85 (1.01-3.27, p=0.038) |
| Abbreviations: BMI: body mass index; CRP: C-reactive protein; CT: chemotherapy; LDH: lactate dehydrogenase; ECOG PS: Eastern Cooperative Oncology Group Performance Status; NA: not available; TE: thromboembolic event; WBC: white blood cells. | | | |

**Supplementary Figure 1.** Receiver Operator Characteristics (ROC) curve for logistic regression model for thromboembolic events. The Harrell's c-index, corresponding to the area under the ROC was 0.78.

**
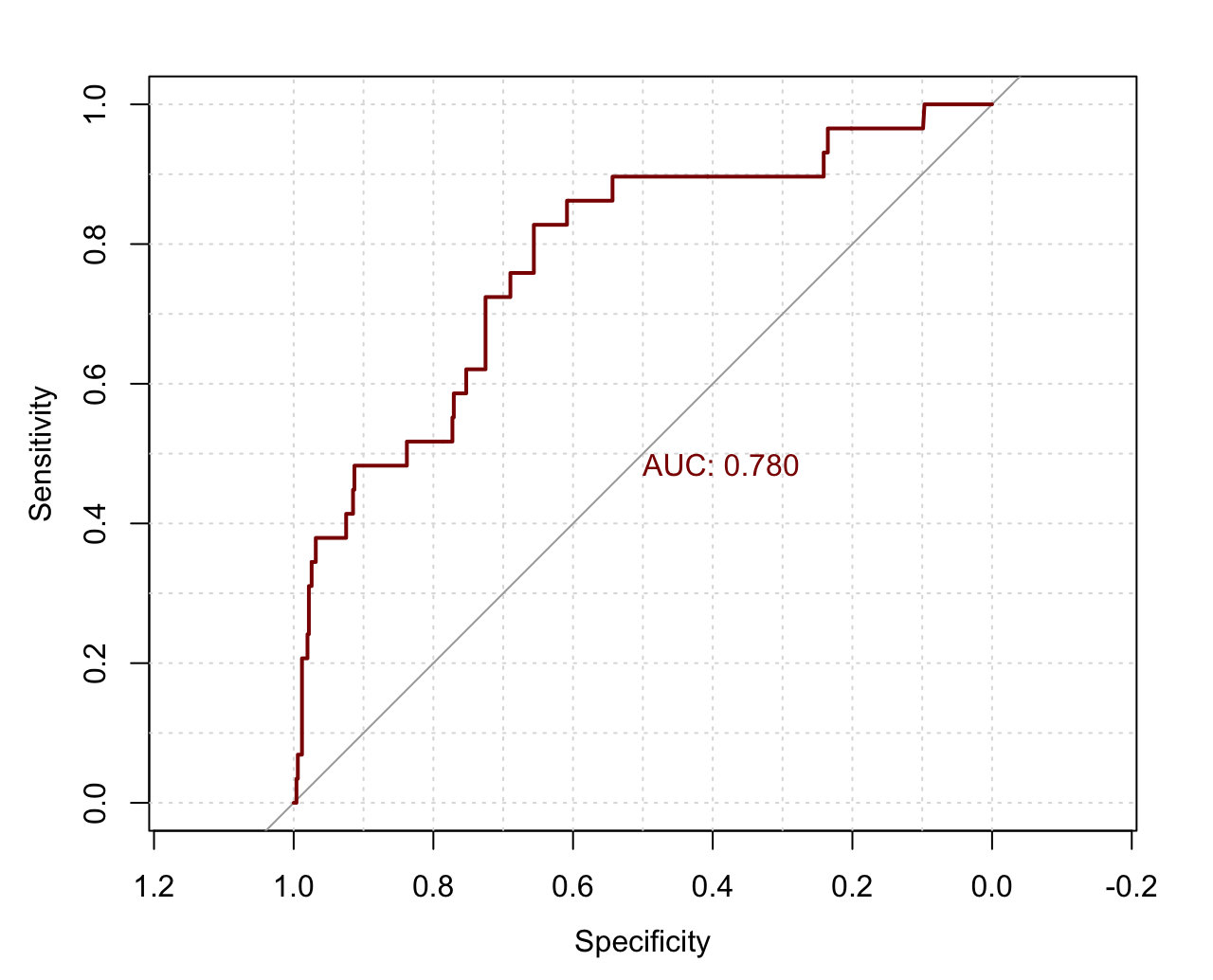
**

**Supplementary Figure 2.** Calibration plot of the weighted TE risk score. Cases are grouped in 10 quantiles, with overall distribution plotted below. *Abbreviations*: TE: thromboembolic event.

**
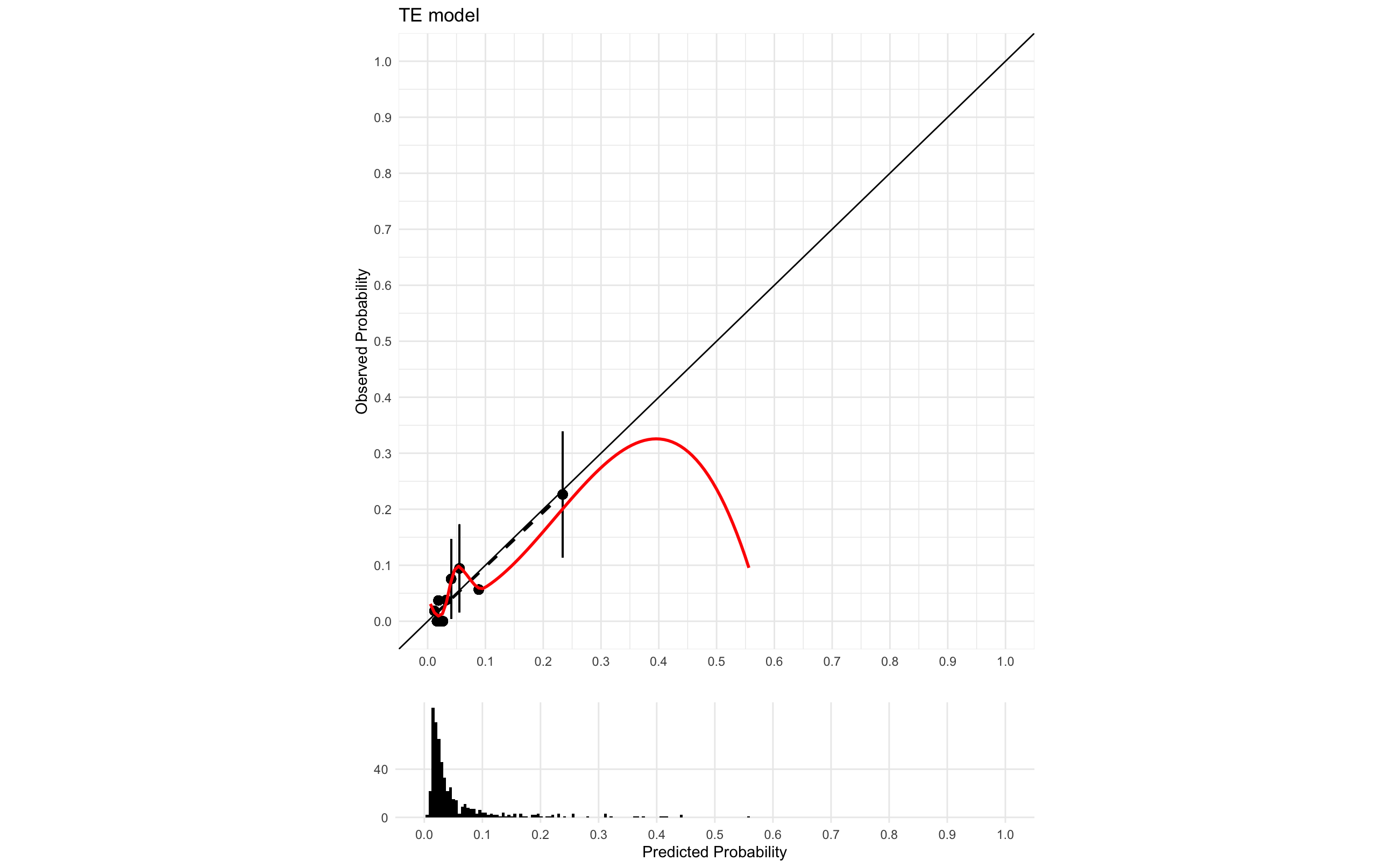
**

**Supplementary Figure 3.** Decision curve analysis. *Abbreviations: KS: Khorana Score.*


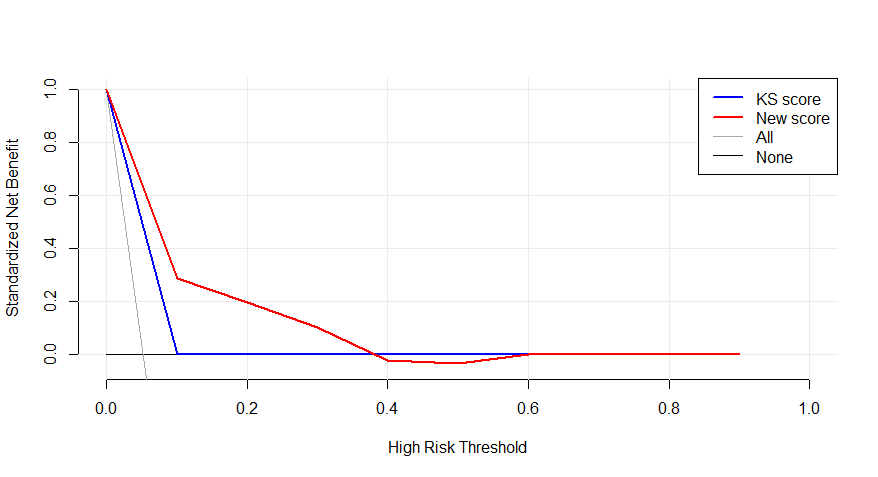


**Supplementary Figure 4.** Landmark analysis of Overall Survival according to the occurrence of TEs during or after hospitalization. OS was calculated from the 45^th^ day after discharge until death or last follow up. *Abbreviations:* NA: not available (not reached), OS: Overall survival TEs: thromboembolic events.
